# Causal Relationship Between the Spread of the COVID-19 and Geopolitical Risks in Emerging Economies

**DOI:** 10.3389/fpubh.2020.626055

**Published:** 2020-12-17

**Authors:** Liangjun Wang, Chunding Li, Xiaohua Chen, Lili Zhu

**Affiliations:** ^1^School of Economics, Zhejiang University of Technology, Hangzhou, China; ^2^College of Economics and Management, China Agricultural University, Beijing, China; ^3^School of Economics and Management, Zhejiang Sci-Tech University, Hangzhou, China; ^4^School of Business, Shenandoah University, Winchester, VA, United States

**Keywords:** COVID-19 pandemic, measuring the spread of the COVID-19, geopolitical risks, emerging economies, panel granger non-causality tests

## Abstract

This study investigates the causality between the spread of the COVID-19 pandemic (measured by new cases per million and new deaths per million) and geopolitical risks (measured by the index of geopolitical risks). We use the balanced panel data framework in 18 emerging economies from January 2020 to August 2020. We run the initial tests of cross-sectional dependence and the panel unit root tests with capturing cross-sectional dependence. Then, we utilize the panel Granger non-causality tests for heterogeneous stationary panel datasets. According to the findings, there is a significant causality from both measures of spreading the COVID-19 pandemic to geopolitical risks. Further tests are performed, and potential implications are also discussed.

## Introduction

The new type of Coronavirus, so-called the COVID-19, emerged in the very late days of 2019 and has affected every corner of the world by providing different aspects. Governments have implemented different policy implications to address the negative consequences of the spread of the COVID-19 pandemic. Lockdowns, closing down public areas, such as public buildings, schools, and various meeting areas, have been the leading measures to slow down the spread of the novel virus (1).

The COVID-19 pandemic has negatively affected the financial markets (2). The COVID-19 pandemic also makes the economies more unstable via various channels, such as the volatility in commodity markets and financial markets. Particularly, emerging economies have experienced significant volatility in their export revenues. Therefore, there is a significant difference between the situation faced by emerging economies and developed countries when facing the COVID-19 pandemic. At this stage, the COVID-19 pandemic creates governance problems, especially in emerging economies, due to the lack of coordination capacity. On the other hand, responding to the pandemic range from very organized in China's case to chaotic Brazil and Mexico. We aim to examine whether these issues affect the geopolitical risks.

This paper aims to examine the causal relationship between the spread of the COVID-19 pandemic (measured by new cases per million and new deaths per million) and geopolitical risks (measured by geopolitical risks index). For this purpose, we use the balanced panel data framework in 18 emerging economies for the period from January 2020 to August 2020. The theoretical relationship between the COVID-19 pandemic and geopolitical risks can be positive or negative. Significant job losses from the COVID-19 have decreased people's income, and this issue may lead to an increase in violence and protests. However, the decline in global demand decreases the value of natural resources, such as oil prices. Then, there should be less conflict over control of these rentable natural resources. For instance, Bloem and Salemi (3) observe that conflicts have increased in some countries (e.g., the Philippines and Nigeria) but decreased in others (e.g., Syria) due to the COVID-19 pandemic. Similarly, Basit (4) indicates that the COVID-19 pandemic has a mixed impact on terrorism. Travel restrictions can decrease terrorism at this stage, but terrorist groups may have a higher capacity to recruit young people from the internet during the lockdown periods.

There are also several previous papers, which have similar researches objectives to our paper. For instance, Sharif et al. (5) show that the uncertainty related to the COVID-19 outbreak has a significant increasing impact on the United States' geopolitical risks. The impact is higher than the impact of uncertainty related to economic policies. However, Apergis and Apergis (6) find that the COVID-19 pandemic decreases the level of political polarization in the United States, measured by the index of partisan conflict, from January 21, 2020, to April 30, 2020. On the other hand, what might indirectly affect the geopolitical risk might be the released confirmed cases' information rather than the case itself. As a result, sentiment and news can also be important in determining the causal relationship between geopolitical risks and COVID-19 (7, 8).

To the best of our knowledge, this is the first research that investigates the causality relationship between the spread of the COVID-19 pandemic and the geopolitical risks in 18 emerging economies. At this stage, we run the initial tests of cross-sectional dependence and the panel unit root tests with capturing cross-sectional dependence. Then, we utilize the panel Granger non-causality test of Dumitrescu and Hurlin (9) for heterogeneous stationary panel datasets. This test procedure captures the heterogeneity and cross-sectional dependence among the emerging economies, which is an important aspect of examining the relationship between the COVID-19 spread and geopolitical risks. Since this test methodology also uses the bootstrapped critical values, the results are robust to the size distortion, which may be a possible issue in the relatively short period of the COVID-19 pandemic. The findings show that the significant causality from both measures of spreading the COVID-19 pandemic to geopolitical risks. Further tests are performed to check the validity of the baseline findings.

The rest of the study is structured as follows. Section Model, Data, and Estimation Procedure introduces the estimated models, the data, and the estimation methodology. Section Empirical Findings provides the empirical findings with further tests on the baseline findings. Section Conclusion concludes.

## Model, Data, and Estimation Procedure

### Estimated Models

We consider below empirical models, which are estimated by the Granger non-causality test procedures, for heterogeneous panel datasets:

(1) $$\begin{array}{r} \Delta NCPM_{i,t}=\alpha_{1}+\alpha_{2}GPR_{i,t-1}+\varepsilon_{i,t} \end{array}$$

(2) $$\begin{array}{r} \Delta NDPM_{i,t}=\alpha_{3}+\alpha_{4}GPR_{i,t-1}+\varepsilon_{i,t} \end{array}$$

(3) $$\begin{array}{r} GPR_{i,t}=\alpha_{5}+\alpha_{6}\Delta NCPM_{i,t-1}+\varepsilon_{i,t} \end{array}$$

(4) $$\begin{array}{r} GPR_{i,t}=\alpha_{7}+\alpha_{8}\Delta NDPM_{i,t-1}+\varepsilon_{i,t} \end{array}$$

In Equations (1–4), where Δ*NCPM*_*i,t*_ and Δ*NCPM*_*i, t*−1_ are the current and the lagged changes of new COVID-19 cases per million people in an emerging country *i* at *t* and *t–1*. Besides, Δ*NDPM*_*i,t*_ and Δ*NDPM*_*i, t*−1_ are the current and the lagged changes of new COVID-19 deaths per million people in an emerging economy *i* at *t* and *t–1*. Finally, *GPR*_*i,t*_ and *GPR*_*i, t*−1_ are the current and lagged geopolitical risks in an emerging country *i* at *t* and *t–1*. Note that the error term is represented by ε_*i,t*_.

### Data

In this study, we estimate the models from Equations (1–4) to examine the causality between the spread of the COVID-19 and geopolitical risks. The sample focuses on the period from January 2020 to August 2020. We include the balanced panel dataset in 18 emerging economies: Argentina, Brazil, China PR, Colombia, India, Indonesia, Israel, Korea Republic, Malaysia, Mexico, the Philippines, Russia, Saudi Arabia, South Africa, Thailand, Turkey, Ukraine, and Venezuela. The countries' selection and the starting period of the empirical analyses are based on the data's availability. The frequency of the sample is monthly.

The spread of the COVID-19 is measured by two indicators: new COVID-19 cases per million people and new COVID-19 deaths per million people. These data are obtained by the dataset of Hasell et al. (10), and they are downloaded from the *World in Data COVID-19* dataset (https://github.com/owid/covid-19-data/tree/master/public/data). We consider the cases per million people to capture countries' size in the spread of the COVID-19 pandemic (11).

Geopolitical risks are measured by the index of geopolitical risks (GPR). The data and the estimation procedure of the country-specific GPR indices are introduced by Caldara and Iacoviello (12). The related data are downloaded from the website of the authors (https://www.matteoiacoviello.com/gpr.htm#dat). A higher value of the GPR index indicates a higher level of geopolitical risks. The GPR index is based on the news related to geopolitical risk. The authors search the archives of 11 international newspapers Boston Globe, Chicago Tribune, Daily Telegraph, Financial Times, Globe and Mail, Guardian, Los Angeles Times, New York Times, Times, Wall Street Journal, and Washington Post. The authors introduce the index by calculating the news related to all news articles related to geopolitical risks. The calculation is the basis of the data at the monthly frequency. Finally, the authors normalize the values and define a benchmark value as an average of 100 for 2000 to 2009. Therefore, a value of 200 in October 2020 means that the GPR level is two-fold higher in October 2020 than the average during 2000–2009.

The GPR index news is based on six groups of searches: The first group of words includes the military-related tensions in leading countries. The second group of words includes nuclear tensions. The third group of words focuses on the articles related to war threats. The fourth group of words includes the news related to terrorist threats. The fifth and sixth words are based on the articles related to actual events, such as terrorist acts and active wars. In short, the first, the second, the third, and the fourth group of words are related to geopolitical threats, and the fifth and the sixth group of words are related to the actual geopolitical events (12).

Finally, we report a summary of descriptive statistics for three indicators in the dataset in Table 1.

**Table 1:** Summary of the descriptive statistics.

**Indicator**	**Definition**	**Abbreviation**	**Mean**	**Std. Dev**.	**Min**.	**Max**.	**Obs**.
New COVID-19 cases per million	Number	NCPM	671.0	1335	0.000	6326	144
New COVID-19 deaths per million	Number	NDPM	17.00	37.28	0.000	187.0	144
Geopolitical risks	Index	GPR	98.22	43.82	34.92	243.4	144

*Indicators are provided in 18 emerging economies for the period from January 2020 to August 2020*.

### Estimation Methodology

First, we check the cross-sectional dependence among the panel units for the new COVID-19 cases per million people (*NCMP*), the new COVID-19-related deaths per million people (*NDMP*), and the index of the geopolitical risks (*GPR*). For this purpose, we utilize the Cross-Section Dependence (CD) test of Pesaran (13, 14) to check the series' cross-sectional dependence. Since we reject the null hypothesis that series are not cross-sectionally dependent and obtain the evidence favoring cross-sectional dependence among the variables, we should apply a panel unit root test that captures the effects of cross-sectional dependence in the unit root methodology. In this paper, we run the cross-sectional dependent Im, Pesaran, and Shin (CIPS) panel unit root test of Pesaran (15).

After confirming the stationarity of indicators by following the results of the panel unit root test of Pesaran (15), we utilize the Granger non-causality test of Dumitrescu and Hurlin (9) for heterogeneous panel datasets. The test procedure of the non-causality test of Dumitrescu and Hurlin (9) is based on the simple averages of classical Granger causality test statistics for each panel unit root test (18 emerging economies in our research). The test statistics in this approach is called as the Wbar test statistic. The Wbar test statistic can also be standardized by considering standard normal distribution with the bootstrapped critical values. This test statistic is called the Zbar statistic (16).

## Empirical Findings

### Results of the CD and CIPS Tests

Before the non-causality analysis, we firstly analyze whether there is a significant cross-sectional dependence in the panel units for *NCMP, NDMP*, and *GPR*. For this purpose, we run the Cross-section Dependence (CD) test proposed by Pesaran (13, 14). The related results are reported in Table 2.

**Table 2:** Cross-sectional dependence test of Pesaran (13, 14).

**Test statistics**	**NCPM**	**NDPM**	**GPR**
Cross-sectional dependence test statistics	11.92^***^ [0.00]	12.15^***^ [0.00]	5.024^***^ [0.00]
Scaled Lagrange multiplier test statistics	17.69^***^ [0.00]	21.52^***^ [0.00]	4.874^***^ [0.00]

*Null hypothesis: Series are not cross-sectionally dependent*.

*** *p < 0.01, and the p-values are in brackets*.

The findings in Table 2 provide the Cross-sectional Dependence and Scaled Lagrange Multiplier test statistics for *NCMP, NDMP*, and *GPR*, respectively. The findings indicate that the null hypothesis is that series are not cross-sectionally dependent are rejected at the 1% significance level (*p* < 0.01). In other words, we observe that all panel data series under concern are cross-sectionally dependent. Therefore, we should move on with the second-generation panel unit root test, which captures the panel units' cross-sectionally dependency. We proceed with the CIPS panel unit root test of Pesaran (15), and the related findings are reported in Table 3.

**Table 3:** Panel unit root test of Pesaran (15).

**Panel unit root test (CIPS)**	**NCPM**	**ΔNCPM**	**NDPM**	**ΔNDPM**	**GPR**	**ΔGPR**
Specification without trend	4.646 [0.99]	−3.217^***^ [0.00]	0.913 [0.82]	−4.224^***^ [0.00]	−2 978^***^ [0.00]	−19 17^***^ [0.00]
Specification with trend	4.155 [0.99]	−3.813^***^ [0.00]	2.506 [0.99]	−4.761^***^ [0.00]	−2 594^***^ [0.00]	−21 52^***^ [0.00]

*Null hypothesis: Series are unit root*.

*** *p < 0.01, and the p-values are in brackets*.

The findings in Table 3 report the CIPS test statistics for both specifications without trend and with trend for the series of *NCMP*, Δ*NCMP, NDMP*, Δ*NDMP, GPR*, and Δ*GPR*, respectively. The results state the null hypothesis is that “series are not unit root” rejected at the 1% significance level (*p* < 0.01) for Δ*NCMP*, Δ*NDMP*, and *GPR*. Therefore, we should proceed with the stationary series (Δ*NCMP*, Δ*NDMP*, and *GPR*) by running the panel Granger non-causality test of Dumitrescu and Hurlin (9), which can successfully model the cross-sectional dependence in the stationary panel units. This evidence also shows that the related variables cannot be cointegrated (16).

### Results of the Dumitrescu–Hurlin Non-causality Test

The results for panel data Granger non-causality test of Dumitrescu and Hurlin (9) are reported in Table 4.

**Table 4:** Panel data granger non-causality test of Dumitrescu and Hurlin (9).

**Hypothesis**	**W-Stat**	**Zbar-Stat**	**Prob**.
ΔNCPM does not homogeneously cause GPR	4.013^***^	6.661^***^	[0.0019]
GPR does not homogeneously cause ΔNCPM	0.086	0.248	[0.7804]
ΔNDPM does not homogeneously cause GPR	3.905^***^	6.241^***^	[0.0028]
GPR does not homogeneously cause ΔNDPM	1.793	2.109	[0.1266]

*** *p < 0.01, and the p-values are in brackets*.

The findings of the panel data Granger non-causality test of Dumitrescu and Hurlin (9) in Table 4 indicate that there is a statistically significant causality (*p* < 0.01) from both Δ*NCPM* and Δ*NDPM* to *GPR*. In other words, both ΔNCPM and ΔNDPM homogeneously cause GPR in the panel dataset of 18 emerging economies from January 2020 to August 2020. The W-Stat and the Zbar-Stat test statistics are statistically significant at the 1% level (*p* < 0.01). Furthermore, there is no statistically significant causality from *GPR* to Δ*NCPM* and Δ*NCPM*, according to the W-Stat and the Zbar-Stat test statistics. These findings indicate that the spread of the COVID-19 causes geopolitical risks in emerging economies. Next, we do several further tests to enhance the implications.

### Further Tests

We also implement several further tests to provide the robustness of the findings and to enhance the implications. The related results are not reported due to the page constraints, but they are available upon request.

Firstly, note that Dumitrescu–Hurlin test statistics do not show whether the coefficients of causal relationships are positive or negative in the model estimations (17). At this stage, we run the fixed-effects estimations to examine the coefficients of the effects of the spread of the COVID-19 on geopolitical risks. Theoretically speaking, the spread of the COVID-19 should increase the level of geopolitical risks and terrorism in emerging economies (18). We observe the positive effects of the spread of the COVID-19 on geopolitical risks in 18 emerging economies.

Secondly, there can be a possible omitted variable bias due to the first-differenced nature of Δ*NCPM* and Δ*NDPM*, given that our causality analysis also includes two variables. Therefore, we both include Δ*NCPM* and Δ*NDPM* together and examine their effects on geopolitical risks. We confirm that the spread of the COVID-19 increases the level of geopolitical risks in 18 emerging economies.

Thirdly, we consider different lags. We automatically define the lag structure as one lag, but the results may be changed regarding the lag length. Given that we have relatively short periods, we consider different lag selection criteria. The baseline findings do not change significantly.

Finally, there are some zero values in the sample, particularly most of emerging economies in January 2020 and February 2020. We exclude the zero values from the sample and re-estimate the causality analysis. When we exclude the zero values, we can also use both logs of Δ*NCPM* and Δ*NDPM*. At this stage, we also re-estimate the causality analysis by the natural logarithmic values of Δ*NCPM*, Δ*NDPM*, and the *GPR* index.

All results are robust to consider these issues in the causality analyses. Overall, we conclude that the spread of the COVID-19 increases the level of geopolitical risks in 18 emerging economies.

## Conclusion

This paper examined the causal relationship between the spread of the COVID-19 pandemic and geopolitical risks. The spread of the COVID-19 is measured by new cases per million and new deaths per million. The geopolitical risks are captured by the index of the GPR. At this stage, we focused on the balanced panel data of 18 emerging countries over the period January 2020-August 2020. Firstly, we applied the tests of Cross-sectional Dependence of Pesaran (13, 14) and the panel unit root test of Pesaran (15) with capturing cross-sectional dependence. Following these tests' results, we implemented the panel Granger non-causality tests of Dumitrescu and Hurlin (9) for heterogeneous panel datasets.

The geopolitical conflicts in emerging economies may divert people's attention from the government's ineffective response to the COVID-19, or it may be the country's use of the health crisis of neighboring countries or the decline of national strength to gain benefits. In this paper, we observed a significant causality from both measures of the spread of the COVID-19 pandemic to geopolitical risks. This evidence indicates that the spread of the COVID-19 pandemic can lead to significant issues in emerging economies related to geopolitical risks. Lockdowns or other implications for slowing down the spread of the COVID-19 virus can also help emerging economies decrease geopolitical risks. Future papers on this subject can focus on specific cases of geopolitical issues, such as terrorism or civil unrest, to analyze the potential effects of the COVID-19 pandemic. Various studies can be conducted on the developments related to the COVID-19, especially in terms of geopolitical risks in each developing country.

## Data Availability Statement

Publicly available datasets were analyzed in this study. This data can be found here: https://github.com/owid/covid-19-data/tree/master/public/data; https://www.matteoiacoviello.com/gpr.htm#dat.

## Author Contributions

LW: data curation and writing—original draft preparation. CL: writing—original draft preparation. XC: conceptualization and investigation. LZ: software and visualisation. All authors contributed to the article and approved the submitted version.

## Conflict of Interest

The authors declare that the research was conducted in the absence of any commercial or financial relationships that could be construed as a potential conflict of interest.
